# Analysis of the vaccination situation against Mpox in people living with HIV/AIDS: an ecological study

**DOI:** 10.1590/0034-7167-2023-0234

**Published:** 2024-10-25

**Authors:** Núbia Vanessa da Silva Tavares, Amuzza Aylla Pereira dos Santos, Mariana Maria Pereira Cintra Farias Carvalho, Kariane Omena Ramos Cavalcante de Melo, Hillary Gabriela dos Santos Oliveira, Thaís Honório Lins Bernardo, Selma Sabrina de Albuquerque Calheiros, José Augustinho Mendes Santos

**Affiliations:** I Universidade Federal de Alagoas. Maceió, Alagoas, Brazil; II Universidade de Brasília. Brasília, Distrito Federal, Brazil

**Keywords:** Vaccine, Monkeypox, Vaccination Coverage, HIV, AIDS, Vacuna, Viruela del Simio, Cobertura de Vacunación, VIH, SIDA

## Abstract

**Objectives:**

to analyze the vaccination situation against Mpox in people living with HIV/AIDS in Brazil.

**Methods:**

an ecological study on the vaccination status against Mpox in people living with HIV/AIDS (PLWHA) in Brazil. The data were collected in April 2023 through information from the Ministry of Health, using the “Microsoft app Power BI,” which is publicly accessible.

**Results:**

the data analysis revealed that in Brazil, 2,978 doses of the MVA-BN Jynneos Mpox vaccine were administered in PLWHA, resulting in a vaccination coverage of 18.3%, with the southern and southeastern regions showing the lowest and highest vaccination coverage, respectively. Gender-based evaluation showed a higher proportion of vaccinated males.

**Conclusions:**

we identified low vaccination coverage in all regions of Brazil, highlighting the need for intensified vaccination activities, especially for PLWHA.

## RESUMEN

**Objetivos:**

analizar la situación de vacunación contra la Mpox en personas que viven con VIH/sida en Brasil.

**Métodos:**

estudio ecológico sobre la situación de vacunación contra la Mpox en personas que viven con VIH/sida (PVVS) en Brasil. Los datos se recopilaron en abril de 2023 a través de información del Ministerio de Salud, denominada “*Microsoft app Power BI”,* de acceso público.

**Resultados:**

el análisis de los datos reveló que se administraron en Brasil 2,978 dosis de la vacuna MVA-BN Jynneos Mpox en PVVS, lo que corresponde a una cobertura de vacunación del 18.3%, siendo las regiones sur y sudeste las que presentaron la menor y mayor cobertura de vacunación, respectivamente. La evaluación por género mostró una mayor proporción de vacunados del género masculino.

**Conclusiones:**

identificamos bajas tasas de cobertura de vacunación en todas las regiones de Brasil, lo que destaca la necesidad de intensificar las actividades de vacunación, especialmente para las PVVS.

## INTRODUCTION

Monkeypox (Mpox) is a zoonotic disease caused by the Mpox virus, primarily found in Central and West African countries. It presents symptoms similar to smallpox, including fever, body rashes, and lymph node swelling, albeit with less severity. Regarding the disease’s transmission among humans, it is observed that it occurs through contact with bodily fluids, skin lesions, mucous membranes, and respiratory droplets, with its severity directly related to the individual’s immune status^(1)^.

In this context, when there is an escalation of cases that could lead to increased morbidity and mortality rates among vulnerable groups, such as people living with HIV/AIDS (PLWHA), strategies to mitigate risks for this population become crucial. In this perspective, it is known that one of the proposed strategies involves the restructure and advancement of care for PLWHA, aiming to enhance their quality of life and life expectancy, reshaping the disease profile and characterizing it as a chronic condition. Furthermore, the physiopathological characteristics of HIV/AIDS infection lead to immunodeficiency, placing these individuals in situations of vulnerability to acquiring other viral, fungal, and/or bacterial infections, thereby increasing the risk of mortality^(2)^.

The vulnerability that PLWHA face extends across social, economic, political, cultural, and educational contexts, as well as opportunities for access to health promotion and disease prevention policies and programs. In this regard, the National Immunization Program (PNI), through vaccination initiatives, emerges as a Public Policy aiming to ensure universality, comprehensiveness, and equity in healthcare for human populations, being one of the most important programs for reducing morbidity and mortality from vaccine-preventable diseases^(3)^.

Given the current epidemiological scenario, with the emergence of the Mpox virus, the World Health Organization (WHO) has made efforts to prevent infection and reduce transmission chains, including the production of the MVA-BN Jynneos Mpox vaccine (Bavarian Nordic Smallpox Vaccine) for emergency use in specific populations, including PLWHA, due to their higher risk of illness and severe disease development. Additionally, it has been noted that, on a global scale, the majority (84.4%) of confirmed Monkeypox cases have occurred in men who have sex with men, with 48% of cases in HIV-positive individuals^(4)^.

From the epidemiological analysis in Brazil, it was observed that in April, there was a 1.2% increase in Mpox notifications. Nevertheless, there has been a downward trend in the number of reported cases classified as confirmed and probable over the last five months. Of these cases, 45.3% occurred in PLWHA, justifying the recommendation to initiate vaccination in this population. Specifically, cisgender men, transgender women, and transgender women aged 18 and above, especially those with a CD4 T-lymphocyte count of less than 200 cells in the last 6 months, began receiving the vaccine in March 2023^(1, 4, 5, 6)^.

In light of the above, considering the indispensability of vaccination with MVA-BN Jynneos Mpox to protect PLWHA against the Mpox virus, the following guiding question arises: What is the vaccination status against Mpox among PLWHA in Brazil?

## OBJECTIVES

To analyze the vaccination status against Mpox in people living with HIV/AIDS in Brazil.

## METHODS

### Ethical Considerations

This study was conducted in accordance with the ethical principles established in Resolution No. 510/16 of the National Health Council^(7)^, which deals with the applicable regulations for research. Since this involves methodological procedures involving research in databases, with aggregated information and no possibility of individual identification, the data were obtained indirectly from publicly accessible information. Therefore, it does not pose risks or identification to the study population.

### Study Design, Location, Population, and Period

This is an ecological study. The study population consisted of PLWHA with a CD4 T-lymphocyte count of less than 200 cells in the last 6 months, as defined in the technical document issued by the Ministry of Health^(1)^.

### Study Protocol

Data collection was conducted in April 2023 and was obtained from the information available on the Ministry of Health’s website, called the “Microsoft app Power BI”, which is publicly accessible and available at the following web address: https://bit.ly/3tjbUwk.

These data represent the absolute number of doses administered for the Mpox vaccine in PLWHA in Brazil. The reporting procedure for this study followed the STROBE guidelines^(8)^.

The collected data were organized according to the following variables: doses administered by region (North, Northeast, Midwest, South, and Southeast), federal unit (UF), doses administered (D1: 1^st^ dose and D2: 2^nd^ dose), gender (female; male), and age group (18 to 80 years or more). The age group used for analysis was determined based on the age recommendations for vaccination in this population, as established by the Ministry of Health, i.e., individuals aged 18 years or older.

### Results Analysis and Statistics

The obtained numbers were tabulated and compiled using Microsoft Excel 2016 for Windows®, and subsequently subjected to descriptive analysis. The analysis of the vaccination situation was conducted by calculating vaccination coverage by UF and region. Vaccination coverage was calculated for the 5 regions and the 26 states of Brazil, plus the Federal District, using the formula adopted by the Ministry of Health for calculating vaccination coverage, which is as follows: the total of the last doses of the primary vaccination schedule (D2) divided by the target population, which in this study is the number of people living with HIV/AIDS eligible for vaccination, multiplied by 100, as described below^(9)^:

$$\frac{\text{Total of last doses administered}\left({\text{D}}_{2}\right)\text{of the Mpox vaccine in PLWHA}}{\text{Number of PLWHA}}\times 100$$

The total number of PLWHA eligible to receive the vaccine was obtained from the Operational Technical Report on Mpox Vaccination from the Ministry of Health, which considered the number of PLWHA who underwent at least one CD4 test in the last six months of 2022 in the public health system and whose last test result during this period was less than 200 cells/mm^(1)^. Additionally, absolute and relative frequencies were determined to analyze the proportion of vaccinated individuals according to doses administered by region of Brazil, the coefficient of doses administered by gender and region of Brazil, and the proportion of vaccinated individuals by gender and age group.

## RESULTS

**Figure 1:**
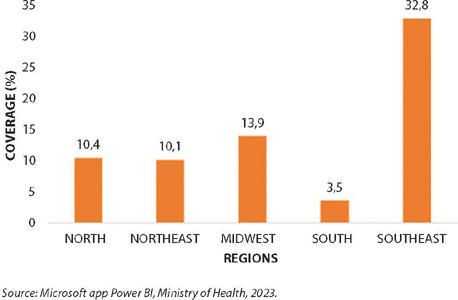
Vaccination coverage of MVA-BN Jynneos Mpox in people living with HIV/AIDS by region, Brazil, 2023

*Source: Microsoft app Power BI, Ministry of Health, 2023.*

The analysis of the data revealed that, up to this point, a total of 2,978 doses of the MVA-BN Jynneos Mpox vaccine have been administered in Brazil to people living with HIV/AIDS (PLWHA), corresponding to a vaccination coverage of 18.3%. The evaluation, broken down by health region, shows variation in the percentage of vaccination coverage, with the southern and southeastern regions having the lowest and highest coverage, respectively (3.5% and 32.8%), as described (Figure 1).

**Figure 2:**
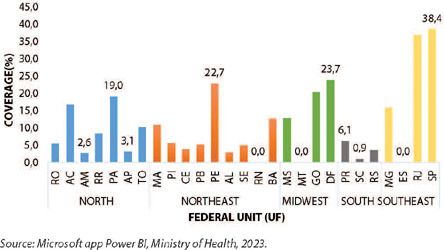
Vaccination coverage of MVA-BN Jynneos Mpox in people living with HIV/AIDS by region and federal units, Brazil, 2023

*Source: Microsoft app Power BI, Ministry of Health, 2023.*

When conducting an individual analysis by federal unit (UF), highlighting the states with the highest and lowest vaccination coverage within each region, it is observed that in the North region, the states of Amazonas and Pará had the lowest and highest percentages of MVA-BN Jynneos Mpox vaccine coverage, respectively (2.6% and 19%). In the Northeast, the states of Rio Grande do Norte and Pernambuco had (0.0% and 22.7%), in the Midwest, the states of Mato Grosso and the Federal District had (0.0% and 23.7%), in the South, Santa Catarina and Paraná had (0.9% and 6.1%), and in the Southeast, the states of Espírito Santo and São Paulo had (0.0 and 38.4%), as described (Figure 2).

**Figure 3:**
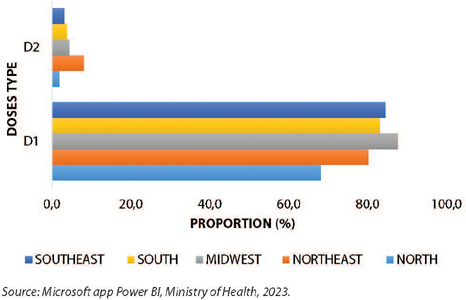
Proportion of doses administered for the MVA-BN Jynneos Mpox vaccine in people living with HIV/AIDS by dose type (D1;D2) by region, Brazil, 2023

*Source: Microsoft app Power BI, Ministry of Health, 2023.*

Regarding the proportion of doses administered by region according to the type of dose (D1;D2), there is a noticeable discrepancy between the proportion of 1st doses administered and 2nd doses for completing the vaccination schedule in all regions of the country. The North region has the lowest proportions for both D1 and D2, as described (Figure 3).

**Figure 4:**
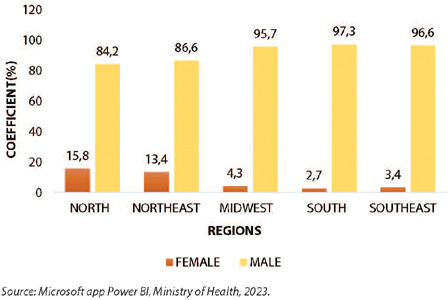
Coefficient of doses administered for the MVA-BN Jynneos Mpox vaccine in people living with HIV/AIDS by gender by region, Brazil, 2023

*Source: Microsoft app Power BI, Ministry of Health, 2023.*

In the comparative analysis by gender, female or male, by region of Brazil, it is noticeable that in all regions, considering the total doses administered to date in PLWHA, there was a predominance of males, with the greatest gender disparity in the southern region (97.3% and 2.7%), as described (Figure 4).

**Figure 5:**
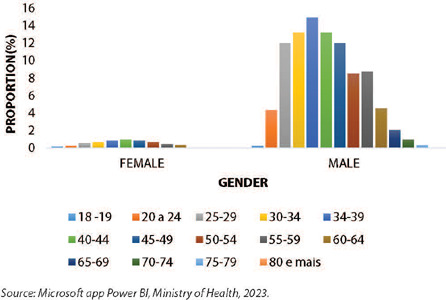
Proportion of people living with HIV/AIDS vaccinated with MVA-BN Jynneos Mpox by gender and age group, Brazil, 2023

*Source: Microsoft app Power BI, Ministry of Health, 2023.*

Comparing the coefficient of vaccinated individuals by gender and age group, for both females and males, the age group with the lowest proportion of vaccinated individuals was between 18-19 years old (0.1% and 0.2%, respectively). Analyzing the female and male categories separately, for females, the age group between 40-44 years had the highest proportion (0.9%) of vaccinated individuals, while for males, this result was concentrated in the 34-39 age group (14.9%). It is important to highlight that for both genders, there were doses administered to the elderly population (starting from 60 years), and in the male population, the proportion of vaccinated individuals in the 60-64 age group was similar to that of the 20-24 age group (4.5% and 4.3%, respectively), decreasing as the age group increases. There have been no doses administered to individuals aged 80 and older for both genders (0.0%), as described (Figure 5).

## DISCUSSION

In our study, across all five regions of Brazil (North, Northeast, Midwest, Southeast, and South), vaccination coverage for the PLWHA group remains very low, despite specific and well-defined vaccination criteria for this population^(1)^. Overall, studies focusing on vaccination topics, including vaccination coverage against HPV and Hepatitis B in PLWHA, have also revealed low vaccination rates, with only a few individuals completing the full vaccination schedule^(10)^. As a result, there is a deficit in vaccination coverage within this population, alongside a reduced rate of seroconversion in this group^(11)^.

In this context, studies dedicated to comprehending the factors contributing to low vaccination coverage typically describe a lack of information about vaccines, fear of potential side effects, and challenges in accessing healthcare services as the primary factors, although they are not the sole reasons behind the low vaccination rates^(12, 13, 14)^. This reality is also observed in Latin American and Caribbean countries^(15)^. Furthermore, the constant spread of Fake News associated with the anti-vaccine movement has significantly contributed to reduced demand for vaccination or the refusal of vaccination services, which, in turn, has impacted vaccination coverage and heightened the discussion regarding the importance of information as a valuable tool for healthcare reform^(16, 17, 18)^.

When assessing the proportion of vaccinated individuals and vaccination coverage by region and UFs (federal units), the study indicates that the Southeast region has the highest proportions. Among the UFs, the state of São Paulo stands out for having a higher percentage of vaccinated individuals but also reports the highest number of confirmed cases of the disease^(4)^. This could be attributed to São Paulo’s larger population, availability of tests, resources for diagnosis, and other contributing factors^(19, 20)^.

When low vaccination coverage rates are observed, this indicator becomes a critical health marker, particularly for vulnerable populations, and serves as a warning sign for the potential introduction or reintroduction of vaccine-preventable diseases^(3)^. In cases where the landscape shows insufficient vaccination coverage against significant diseases like Mpox, it underscores the necessity to proactively engage with vulnerable populations, such as PLWHA, due to their heightened risk of developing severe forms of the disease. This underscores the importance of protective strategies aimed at preventing a significant increase in mortality rates within this population due to severe forms of the disease^(1)^. To sum up, the data emphasize the importance of identifying regions where vaccination coverage falls below recommended levels, with the aim of maintaining continuous and ongoing planning processes, increasing surveillance, and developing timely intervention strategies to reach unvaccinated individuals and sustain a safe epidemiological environment^(19)^.

Regarding the coefficient of doses administered by dose type, there is a noticeable gap between D1 and D2, indicating an incomplete vaccination schedule. Authors point out that a lack of awareness about the importance of vaccination, lack of interest, fear of intramuscular injections, limited time, and fear of side effects are among the primary reasons contributing to this phenomenon^(21, 22)^. However, it cannot be conclusively stated in this study whether these are cases of abandoning the vaccination schedule or if the 30-day interval between doses, as recommended by the Ministry of Health, directly or indirectly affects this trend, as the data analysis had not been completed by the study date. This is because the provided data does not allow for specifying the start date of the vaccination schedules.

Regarding the analysis of the proportion of vaccinated individuals by gender, females in all regions of Brazil had a vaccination rate over 60% higher than males. However, a study that evaluated vaccination and associated factors in both genders obtained different results, as 65% of women had a complete vaccination schedule^(23)^. This difference can be explained by the fact that females culturally seek healthcare services more frequently than males. This behavior may be associated with theoretical-sociocultural constructs that differentiate male and female behaviors, leading to differing attitudes regarding seeking and using healthcare services^(24, 25)^.

Regarding the proportion of doses administered by gender and age group, the male elderly population in the 60-64 age group stands out, with a vaccination rate similar to that of males in the 20-24 age group. This phenomenon can be explained by the results of studies characterizing the profile of elderly individuals living with HIV/AIDS in Brazil, where the majority are male and between 60 and 69 years old^(26, 27)^. Furthermore, among males overall, the highest vaccination rates were concentrated in the 34-39 age group, which is consistent with the distribution of confirmed Mpox cases by gender and age group, where a higher frequency is observed in this gender between 30 and 34 years^(4)^.

It is important to emphasize that the success of the vaccination process for any disease or condition is linked to the acceptance, promotion, and support of healthcare professionals, especially nurses. This is because nurses are facilitators and co-responsible for adherence to vaccination schedules, as vaccination activities are part of their professional duties. Additionally, care is at the core of their activities, and they play a vital role in executing health promotion, protection, and recovery actions based on evidence-based information and aligned with the principles and guidelines of the Unified Health System (SUS). This ensures that the population understands the importance of vaccination for maintaining life^(28)^.

### Study Limitations

It is important to highlight some significant limitations in our research. First, the nature of the event under study, which is still ongoing (the Mpox outbreak), implies that the information is constantly being updated. This includes vaccination adherence, which may lead to variations in vaccination coverage assessed at a later time.

### Contributions to the Nursing, Health, or Public Policy Field

This study contributes to the reflection of healthcare professionals, especially those in the nursing team, regarding the existing gaps in the vaccination process in general and, specifically, in the case of Mpox vaccination in PLWHA. The nursing team plays a direct role in organizing and implementing vaccination actions. Furthermore, it provides focused planning perspectives for managers and healthcare professionals, enabling the implementation of effective interventions to reach the entire target audience, ensuring universal, equal, and equitable access to vaccination, and consequently, reducing the formation of susceptible groups and disease-related morbidity and mortality. This will have a direct impact on the Brazilian epidemiological landscape.

## CONCLUSIONS

We identified low vaccination coverage rates for PLWHA in all regions of Brazil. Among them, the southern region had the lowest vaccination coverage, and the states of Rio Grande do Norte, Mato Grosso, and Espírito Santo recorded zero coverage. Furthermore, there is a need to intensify vaccination activities, especially for PLWHA, and also to increase adherence in both genders to complete the vaccination schedule to prevent the formation of susceptible groups. It is likely that the provision of additional data by the system will allow for an expanded evaluation of the data, including new indicators to analyze other variables related to the vaccination status of PLWHA in Brazil.

It is important to emphasize that this study enabled a critical and detailed analysis of vaccination coverage in different regions of the country, as well as highlighted the need to improve public policies that address the epidemiological changes that have occurred and promote better living and health conditions for this population, thus contributing to minimizing the harm caused by this disease. Considering the vaccination coverage perspective, it is crucial to implement actions aimed at expanding assistance to PLWHA, providing a basis for a discussion on healthcare practice related to vaccination and its peculiarities in the context of vulnerable populations, aiming not only at prevention but also at improving the quality of care.

## References

[1] Ministério da Saúde (BR), Secretaria de Vigilância em Saúde e Ambiente, Departamento do Programa Nacional de Imunizações (2023). Informe Técnico Operacional De Vacinação Contra a Mpox [Internet].

[2] Gerin L, Antonini M, Santos KDS, Gir E, Reis RK (2022). O conhecimento dos profissionais de saúde sobre vacinação de pessoas vivendo com HIV: uma revisão integrativa. Esc Anna Nery.

[3] Domingues CMAS, Maranhão AGK, Teixeira AM, Fantinato FF, Domingues RA (2020). 46 anos do Programa Nacional de Imunizações: uma história repleta de conquistas e desafios a serem superados. Cad Saúde Pública.

[4] Ministério da Saúde (BR), Secretaria de Vigilância em Saúde e Ambiente (2023). Boletim Epidemiológico Especial MPOX nº 21: Boletim Mensal do Centro de Operações de Emergência (COE)[Internet].

[5] Ministério da Saúde (BR), Secretaria de Vigilância em Saúde e Ambiente (2023). Boletim Epidemiológico Especial MPOX nº 22: Boletim Mensal do Centro de Operações de Emergência (COE)[Internet].

[6] Ministério da Saúde (BR), Secretaria de Vigilância em Saúde e Ambiente, Departamento de Imunização e Doenças Imunopreveníveis, Coordenação-Geral de Incorporação Científica e Imunização (2023). Nota Técnica Nº 13/2023-CGICI/DIMU/SVSA/MS[Internet].

[7] Conselho Nacional de Saúde (CNS) (2016). Resolução n.510 de 7 de abril de 2016, dispõe sobre as normas aplicáveis a pesquisas em Ciências Humanas e Sociais cujos procedimentos metodológicos envolvam a utilização de dados diretamente obtidos com os participantes ou de informações identificáveis ou que possam acarretar riscos maiores do que os existentes na vida cotidiana[Internet].

[8] Ebrahim S, Clarke M (2007). STROBE: new standards for reporting observational epidemiology, a chance to improve. Int J Epidemiol.

[9] Ministério da Saúde (BR), Secretaria de Vigilância em Saúde, Departamento de Articulação Estratégica de Vigilância em Saúde (2022). Guia de Vigilância em Saúde [Internet].

[10] Pimenta PDC, Bani GMDAC, Júlio RS (2020). Cobertura vacinal de HPV em pessoas vivendo com hiv/aids[Dissertação] [Internet].

[11] Oliveira EH, Silva ACS, Rêgo IV, Lopes TBC, Rocha KGL, Guimarães LO (2021). Vacinação contra hepatite B em pessoas vivendo com HIV no Estado do Piauí, Brasil. RSD.

[12] Almeida RCAA, Castro JM, Oliveira TVC, Oliveira TF, Araújo DA, Alencar NPFC (2020). Cobertura vacinal ANTI-HPV e motivos de não vacinação. REAEnf.

[13] Padilha ARN, Resende MAA, Reis MD, Oliveira VC, Oliveira PP, Carvalho NM (2022). Motivos para pais e responsáveis pela não adesão à vacinação contra o Papiloma Vírus Humano: scoping review. RSD.

[14] Moreira KCC, Sousa Martins RA (2020). A não vacinação dos filhos e a literacia para a saúde. Rev Fam, Ciclos Vida Saúde Contexto Soc[Internet].

[15] Guzman-Holst A, De Antonio R, Prado-Cohrs D, Juliao P (2020). Barriers to vaccination in Latin America: a systematic literature review. Vaccine.

[16] Saraiva LJC, FARIA JF (2019). A Ciência e a Mídia: a propagação de fake news e sua relação com o movimento anti-vacina no Brasil. Intercom [Internet].

[17] Cardoso VMVS, Bianco E, Accordi NQ, Pimentel ÁBNM, Lourenço FS, Cressoni VD (2021). Vacinas e movimentos antivacinação: origens e consequências. REAC.

[18] Passos FT, Moraes IM (2020). Movimento antivacina: revisão narrativa da literatura sobre fatores de adesão e não adesão à vacinação. Rev JRG.

[19] West AMM, Pacheco TO, Lopes IMD (2023). Vaccination coverage in children under 1 year of age: an analysis between different regions of Brazil. RSD.

[20] Araújo AVS, Oliveira MCNI, Almeida MS, Smith NA (2020). Análise da cobertura da vacina meningocócica c conjugada de 2012 a 2018. Braz J Health Rev.

[21] Silva AR, Leite DS (2021). Cobertura vacinal de adolescentes, adultos e idosos em Marabá (PA), no período de 2015 a 2020. RSD.

[22] Silva LEL, Oliveira MLC, Galato D (2019). Receptividade à vacina contra o papilomavírus humano: uma revisão sistemática Rev Panam Salud Publica.

[23] Araújo TM, Souza FO, Pinho PS (2019). Vacinação e fatores associados entre trabalhadores da saúde. Cad Saúde Pública.

[24] Cobo B, Cruz C, Dick PC (2021). Desigualdades de gênero e raciais no acesso e uso dos serviços de atenção primária à saúde no Brasil. Ciên Saúde Coletiva.

[25] Gomes R, Nascimento EFD, Araújo FCD (2007). Por que os homens buscam menos os serviços de saúde do que as mulheres? as explicações de homens com baixa escolaridade e homens com ensino superior. Cad Saúde Pública.

[26] Maciel M, Ribeiro A, Costa L, Diógenes L, Queiroz D, Farias G (2021). Caracterização sociodemográfica e clínica de idosos vivendo com HIV. CONJ [Internet].

[27] Oliveira DDSD (2019). Caracterização epidemiológica dos casos de HIV/aids em pessoas com 60 anos ou mais, Pará: período 2006 - 2015.

[28] Oliveira GCA, Imperador C, Ferreira ARO, Oliveira WR, Camparoto CW, Jesus WA (2021). Assistência de enfermagem no processo de imunização: revisão de literatura. Braz J Develop.

