# Supplementary material for: Analysis of the vaccination situation against Mpox in people living with HIV/AIDS: an ecological study
Source: Rev Bras Enferm. 2024 Oct 25;77(5):e20230234. doi: 10.1590/0034-7167-2023-0234 (PMC11508629; doi:10.1590/0034-7167-2023-0234)
Supplement: Translation

# Translation: Análise da situação vacinal contra a Mpox em pessoas vivendo com HIV/Aids: estudo ecológico

**DOI:** 10.1590/0034-7167-2023-0234pt

**Authors:** Núbia Vanessa da Silva Tavares, Amuzza Aylla Pereira dos Santos, Mariana Maria Pereira Cintra Farias Carvalho, Kariane Omena Ramos Cavalcante de Melo, Hillary Gabriela dos Santos Oliveira, Thaís Honório Lins Bernardo, Selma Sabrina de Albuquerque Calheiros, José Augustinho Mendes Santos

**Affiliations:** I Universidade Federal de Alagoas. Maceió, Alagoas, Brasil; II Universidade de Brasília. Brasília, Distrito Federal, Brasil

**Keywords:** Vacina, Monkeypox, Cobertura Vacinal, HIV, AIDS

## Abstract

**Objetivos:**

analisar a situação vacinal contra a Mpox em pessoas vivendo com HIV/Aids no Brasil.

**Métodos:**

estudo ecológico sobre a situação vacinal contra a Mpox das pessoas vivendo com HIV/Aids (PVHA) no Brasil. Os dados foram coletados em abril de 2023 através de informações do Ministério da Saúde, denominada “*Microsoft app Power BI”,* de acesso público.

**Resultados:**

a análise dos dados revelou que foram administradas, no Brasil, 2.978 doses da vacina MVA-BN Jynneos Mpox em PVHA, correspondendo a uma cobertura vacinal de 18,3%, sendo as regiões sul e sudeste as que apresentaram a menor e maior cobertura vacinal, respectivamente. A avaliação por sexos mostrou maior proporção de vacinados do sexo masculino.

**Conclusões:**

identificamos baixas coberturas vacinais em todas as regiões do Brasil, expondo a necessidade de intensificação nas atividades de vacinação, sobretudo para as PVHA.

## INTRODUÇÃO

A *Monkeypox* (Mpox) é uma doença zoonótica causada pelo vírus Mpox, cuja endemicidade se concentra em países da África Central e Ocidental, causando sinais e sintomas semelhantes aos da varíola, como febre, erupções corporais e edema de gânglios linfáticos, no entanto, com menor gravidade. Quando se verifica o comportamento da doença para as formas de transmissão entre humanos, observa-se que ocorre por meio do contato com fluidos corporais, lesões cutâneas, mucosas e gotículas respiratórias, estando sua gravidade diretamente relacionada ao status imunológico da pessoa acometida^(1)^.

Nesse contexto, quando se tem um agravamento com proporções que podem ocasionar aumento das taxas de morbimortalidade em grupos vulneráveis, como as pessoas que vivem com HIV/Aids (PVHA), já se pensa em estratégias que possam diminuir os riscos para esta população. Nessa perspectiva, sabe-se que uma das estratégias pensadas foi a reestruturação e o avanço nos cuidados com as pessoas que vivem com HIV/Aids (PVHA), visando ao aumento da qualidade e da expectativa de vida, remodelando o perfil da doença e atribuindo a esta a característica de condição crônica. Além disso, as características fisiopatológicas da infecção por HIV/Aids provocam quadros de imunodeficiência e inserem essas pessoas em situações de vulnerabilidade para a aquisição de outras infecções virais, fúngicas e/ou bacterianas, elevando também o risco de mortalidade^(2)^.

A situação de vulnerabilidade em que as PVHA estão inseridas perpassa contextos sociais, econômicos, políticos, culturais e educacionais, bem como as oportunidades de acesso às políticas e programas de promoção da saúde e prevenção de doenças. Nesse sentido, o Programa Nacional de Imunizações (PNI), através das ações de vacinação, surge como Política Pública que busca garantir a universalidade, a integralidade e a equidade no cuidado aos grupos humanos, sendo um dos programas mais importantes para a redução da morbimortalidade por doenças imunopreveníveis^(3)^.

Diante do cenário epidemiológico atual, com a introdução do vírus Mpox, a Organização Mundial de Saúde (OMS) empreendeu esforços para a prevenção da infecção e redução da cadeia de transmissão, incluindo a produção da vacina MVA-BN *Jynneos Mpox* (Vacina Varíola Bavarian Nordic) para uso emergencial e em populações específicas, nas quais as PVHA foram incluídas devido ao maior risco de adoecimento e desenvolvimento de formas graves da doença. Além disso, verificou-se que, no contexto mundial, a maioria dos casos (84,4%) confirmados de Monkeypox foram em homens que fazem sexo com homens e 48% dos casos em pessoas HIV positivas^(4)^.

Das análises epidemiológicas no Brasil, observou-se que no mês de abril houve um aumento de 1,2% no número de notificações para Mpox. Apesar disso, nota-se uma tendência à redução verificada nos últimos cinco meses dos casos notificados classificados como confirmados e prováveis. Desses casos, 45,3% ocorreram em PVHA, o que também justificou a recomendação para o início da vacinação neste público, ou seja, homens cisgêneros, travestis e mulheres transexuais com idade igual ou superior a 18 anos, especialmente aqueles com contagem de linfócitos T CD4 inferior a 200 células nos últimos 06 meses, tendo seu uso iniciado em março de 2023^(1, 4, 5, 6)^.

Diante do exposto, considerando a indispensabilidade da vacinação com a MVA-BN *Jynneos Mpox* para proteção contra o vírus Mpox nas PVHA, surge a seguinte questão norteadora: qual a situação vacinal contra a Mpox em PVHA no Brasil?

## OBJETIVO

Analisar a situação vacinal contra a Mpox em pessoas vivendo com HIV/Aids no Brasil.

## MÉTODOS

### Aspectos éticos

Este estudo foi conduzido em conformidade com os aspectos éticos estabelecidos na Resolução Nº 510/16 do Conselho Nacional de Saúde^(7)^, que trata das normas aplicáveis à pesquisa. Considerando que se trata de procedimentos metodológicos envolvendo a pesquisa em bancos de dados, com informações agregadas e sem possibilidade de identificação individual, os dados foram obtidos de forma indireta a partir de informações de acesso público. Portanto, isso não acarreta riscos nem identificação para a população em estudo.

### Desenho, local de estudo, população e período

Trata-se de um estudo ecológico. A população estudada consistiu em PVHA com contagem de linfócitos T CD4 inferior a 200 células nos últimos 06 meses, conforme definido no documento técnico emitido pelo Ministério da Saúde^(1)^.

### Protocolo de estudo

A coleta de dados foi realizada no mês de abril de 2023 e foi obtida por meio dos dados disponíveis na página eletrônica do Ministério da Saúde, chamada “*Microsoft app Power BI”* que é de acesso público e está disponível no endereço eletrônico https://bit.ly/3tjbUwk.

Esses dados referem-se ao número absoluto de doses aplicadas da vacina contra a Mpox em PVHA no Brasil. O procedimento de relato deste estudo seguiu as recomendações do STROBE^(8)^.

Os dados coletados foram organizados de acordo com as seguintes variáveis: doses aplicadas por região (Norte, Nordeste, Centro-Oeste, Sul e Sudeste), unidade federada (UF), doses aplicadas (D1: 1ª dose e D2: 2ª dose), sexo (feminino; masculino) e faixa etária (18 a 80 anos ou mais). A faixa etária utilizada para análise foi determinada com base nas recomendações de idade para vacinação desse público, conforme estabelecido pelo Ministério da Saúde, ou seja, pessoas com 18 anos ou mais.

### Análise dos resultados e estatística

Os números obtidos foram tabulados e compilados no *software Microsoft Excel* 2016 por *Windows*®, e posteriormente submetidos à análise descritiva, sendo a análise da situação vacinal realizada através do cálculo das coberturas vacinais por UF e região. Foram calculadas as coberturas vacinais das 05 regiões e dos 26 estados do Brasil mais o Distrito Federal, utilizando-se para isso a fórmula adotada pelo Ministério da Saúde para o cálculo das coberturas vacinais, a saber, o total de últimas doses do esquema vacinai primário (D2), dividido pela população-alvo que neste estudo é o número de pessoas vivendo com HIV/Aids elegíveis para vacinação, multiplicado por IQÜconforme descrito a seguir^(9)^:

$$\frac{\text{Total de últimas doses aplicadas}\left({\text{D}}_{2}\right)\text{da vacina contra a Mpox em PVHA}}{\text{Número de PVHA}}\times 100$$

O total de PVHA elegíveis para receber a vacina foi obtido através do Informe Técnico Operacional de Vacinação contra a Mpox do Ministério da Saúde que considerou o número de PVHA que realizaram pelo menos um exame de CD4 nos últimos seis meses de 2Q22 no sistema público de saúde, e cujo resultado do último exame realizado neste período foi inferior a 2QQ células/mm^(1)^. Além disso foram determinadas as frequências absoluta e relativa para analisar a proporção de vacinados segundo doses aplicadas por região do Brasil, o coeficiente de doses aplicadas segundo sexo e região do Brasil e a proporção de vacinados segundo sexo e faixa etária.

## RESULTADOS

A análise dos dados revelou que até o momento foram administradas, no Brasil, 2.978 doses da vacina MVA-BN *Jynneos Mpox* em PVHA, correspondendo a uma cobertura vacinal de 18,3%. A avaliação segregada por região de saúde demonstra oscilação no percentual de cobertura vacinal, com destaque para as regiões sul e sudeste que apresentaram a menor e maior cobertura respectivamente (3,5% e 32,8%), conforme descrito (Figura 1).

**Figura 1:** Cobertura vacinal da MVA-BN *Jynneos Mpox* em pessoas vivendo com HIV/Aids por região, Brasil, 2Q23

*Fonte: Microsoft app powerbi, Ministério da Saúde, 2023.*

Ao realizar a análise individual por UF destacando, em cada região, os estados com seus extremos de cobertura vacinal, observa-se que na região Norte os estados do Amazonas e Pará apresentaram, respectivamente, o menor e maior percentual de cobertura da vacina MVA-BN *Jynneos Mpox* (2,6% e 19%), no Nordeste os estados do Rio Grande do Norte e Pernambuco (Q,Q% e 22,7%), no Centro Oeste os estados do Mato Grosso e Distrito Federal (Q,Q% e 23,7%), no Sul temos Santa Catarina e Paraná ( Q,9% e 6,1%)e no Sudeste os estados do Espírito Santo e São Paulo (Q,Q e 38,4%), conforme descrito (Figura 2).

**Figura 2:** Cobertura vacinal da MVA-BN Jynneos Mpox em pessoas vivendo com HVI/Aids por região e unidade federada, Brasil, 2Q23

*Fonte: Microsoft app powerbi, Ministério da Saúde, 2023.*

Em relação à proporção de doses aplicadas por região segundo o tipo de dose (D1;D2), é notória a discrepância entre a proporção de 1° doses aplicadas e as 2° doses para a completude do esquema vacinal em todas as regiões do país, sendo a região norte a que apresenta as menores proporções tanto para D1 como para D2, conforme descrito (Figura 3).

**Figura 3:** Proporção de doses aplicadas da vacina MVA-BN Jynneos Mpox em pessoas vivendo com HIV/Aids segundo o tipo de dose (D1;D2) por região, Brasil, 2Q23

*Fonte: Microsoft app powerbi, Ministério da Saúde, 2023.*

Na análise comparativa por sexo, feminino ou masculino, segundo região do Brasil, nota-se que em todas as regiões, considerando o total de doses aplicadas até o momento em PVHA, houve predominância do sexo masculino, com maior disparidade entre sexos na região sul (97,3% e 2,7%), conforme descrito (Figura 4).

**Figura 4:** Coeficiente de doses aplicadas da vacina MVA-BN Jynneos Mpox em pessoas vivendo com HIV/Aids por sexo segundo região, Brasil, 2Q23

*Fonte: Microsoft app powerbi, Ministério da Saúde, 2023.*

Comparando-se o coeficiente de vacinados segundo sexo e faixa etária, tanto para o sexo feminino como para o sexo masculino, a faixa etária com a menor proporção de pessoas vacinadas foi entre 18-19 anos de idade (com 0,1% e 0,2% respectivamente).

Analisando as categorias feminina e masculina, isoladamente, para o sexo feminino a faixa etária entre 40 a 44 anos apresentou a maior proporção (0,9%) de vacinados, já no sexo masculino esse resultado concentrou-se na faixa etária de 34-39 anos (14,9%). Importante destacar que para ambos os sexos houve doses aplicadas na população idosa (a partir de 60 anos), e no sexo masculino a proporção de vacinados na faixa etária de 60-64 anos equiparou-se à de 20-24 anos (4,5% e 4,3% respectivamente), com decréscimo à medida que aumenta a faixa etária, não tendo, até o momento, doses aplicadas no público de 80 anos e mais para ambos os sexos (0,0%), conforme descrito (Figura 5).

**Figura 5:** Proporção de pessoas vivendo com HIV/Aids vacinadas com MVA-BN Jynneos Mpox segundo sexo e faixa etária, Brasil, 2023

*Fonte: Microsoft app powerbi, Ministério da Saúde, 2023.*

## DISCUSSÃO

Em nosso estudo, em todas as cinco regiões do Brasil (Norte, Nordeste, Centro-Oeste, Sudeste e Sul), a cobertura vacinal para o grupo de PVHA ainda é muito baixa, apesar dos critérios específicos e bem delimitados para a vacinação nesta população^(1)^. De maneira geral, os estudos que abordam a temática da vacinação, incluindo a cobertura vacinal contra o HPV e a Hepatite B em PVHA, também evidenciaram baixas coberturas vacinais, sendo poucos os que possuíam esquema completo^(10)^. Observa-se, portanto, um déficit na vacinação dessa população, além de uma reduzida taxa de soroconversão neste grupo^(11)^.

Nessa situação, os estudos que se dedicaram a compreender os fatores relacionados às baixas coberturas vacinais descrevem, de maneira geral, que a falta de informação sobre as vacinas, o medo dos efeitos colaterais e as dificuldades de acesso aos serviços de saúde estão entre os principais fatores, embora não sejam os únicos responsáveis pela baixa cobertura dos processos vacinais^(12, 13, 14)^. Essa realidade também é observada nos países da América Latina e do Caribe^(15)^. Além disso, as *Fake News* divulgadas constantemente e associadas ao movimento anti-vacina têm contribuído substancialmente para a redução da procura ou recusa ao serviço de vacinação, o que impacta na redução das coberturas vacinais e eleva a discussão acerca da informação como um instrumento valioso para a reestruturação da saúde^(16, 17, 18)^.

Ao avaliar a proporção de vacinados e a cobertura vacinal por região e UFs, o estudo demonstrou que a região Sudeste concentra as maiores proporções. Entre as UFs, o estado de São Paulo destacou-se por possuir um maior percentual de vacinados, mas também obteve o maior número de casos confirmados da doença^(4)^. Isso pode ser justificado pelo fato de que São Paulo possui uma maior população, disponibilidade de testes, recursos para realização do diagnóstico, entre outros fatores^(19, 20)^.

Quando se observam os baixos índices de cobertura vacinal, verifica-se que esse indicador tem sido utilizado como um importante marcador de saúde, principalmente para as populações vulneráveis, e como um indicativo do risco de introdução e/ou reintrodução de doenças imunopreveníveis^(3)^. No momento em que o panorama mostra baixas coberturas vacinais contra uma doença importante como a Mpox, surge um alerta sobre a necessidade de buscar ativamente o público vulnerável, como as PVHA, devido ao aumento do risco de desenvolvimento de formas graves da doença. Isso reforça a importância das estratégias de proteção para evitar que tais formas graves aumentem consideravelmente as taxas de mortalidade nessa população^(1)^. Em resumo, os dados obtidos destacam a necessidade de identificar as regiões onde as coberturas vacinais estão abaixo do recomendado, com o objetivo de manter processos contínuos e permanentes de planejamento, aumentando a vigilância e elaborando estratégias de intervenção oportunas para alcançar os indivíduos não vacinados e manter um cenário epidemiológico seguro^(19)^.

Em relação ao coeficiente de doses aplicadas segundo o tipo de dose, é evidente a discrepância verificada entre a D1 e a D2, ou seja, a não conclusão do esquema vacinal. Autores apontam que a falta de conhecimento sobre a importância da vacinação, a falta de interesse, o medo da injeção intramuscular, a falta de tempo e o medo dos efeitos colaterais estão entre os principais motivos relacionados a esse fenômeno^(21, 22)^. No entanto, não é possível afirmar, neste estudo, se são casos de abandono do esquema vacinal ou se o intervalo de 30 dias entre as doses, preconizado pelo Ministério da Saúde, interfere diretamente ou indiretamente, pois a análise dos dados ainda não havia sido concluída até a data do estudo, uma vez que os dados disponibilizados não permitem precisar a data de início dos esquemas.

Quanto à análise da proporção de vacinados por sexo, observou-se que o sexo feminino apresenta uma diferença de mais de 60% em relação ao sexo masculino em todas as regiões do Brasil. No entanto, uma pesquisa que avaliou a vacinação e os fatores associados em ambos os sexos obteve resultados diferentes dos apresentados neste estudo, uma vez que, comparativamente, 65% das mulheres possuíam esquema vacinal completo^(23)^. Analisando o contexto, sabe-se que, culturalmente, as pessoas do sexo feminino procuram os serviços de saúde com mais frequência, e esse comportamento pode estar associado às construções teórico-socioculturais que diferenciam comportamentos femininos e masculinos, provocando atitudes diferenciadas em relação à procura e ao uso dos serviços de saúde^(24, 25)^.

No que diz respeito à proporção de doses aplicadas por sexo e faixa etária, destaca-se a população idosa do sexo masculino na faixa etária de 60-64 anos, que apresentou uma proporção semelhante à aplicada para o mesmo sexo na faixa dos 20-24 anos de idade. Esse fenômeno pode ser explicado pelos resultados de alguns estudos que caracterizaram o perfil de idosos vivendo com HIV/Aids no Brasil, em que a maioria é do sexo masculino e pertence à faixa etária de 6Q a 69 anos de idade^(26, 27)^. Além disso, no sexo masculino, de maneira geral, as maiores proporções concentraram-se na faixa etária de 34-39 anos, o que está alinhado com a distribuição dos casos confirmados de *Mpox* por sexo e faixa etária, onde se observa uma maior frequência neste sexo entre 3Q e 34 anos^(4)^.

É importante ressaltar que o sucesso do processo de vacinação relacionado a qualquer agravo ou doença está associado à aceitação, divulgação e acolhimento dos profissionais de saúde, principalmente os enfermeiros. Isso se deve ao fato de que eles são facilitadores e co-responsáveis pelo processo de adesão aos esquemas vacinais, uma vez que as atividades de vacinação fazem parte das responsabilidades dessa profissão. Além disso, o cuidado está no centro de suas atividades, materializando-se no desenvolvimento de ações de promoção, proteção e recuperação da saúde, com base em informações baseadas em evidências científicas e alinhadas aos princípios e diretrizes do Sistema Único de Saúde (SUS), de forma que a população compreenda a importância da vacinação para a manutenção da vida^(28)^.

### Limitações do estudo

É importante destacar algumas limitações significativas em nossa pesquisa. Primeiramente, a natureza do evento em estudo, que ainda está em curso (o surto de Mpox), implica que as informações estão em constante atualização. Isso inclui a adesão à vacinação, o que pode levar a variações nas coberturas vacinais avaliadas em um momento posterior.

### Contribuições para a área da enfermagem, saúde ou política pública

Este estudo contribui para a reflexão dos profissionais de saúde, especialmente os da equipe de enfermagem, sobre as lacunas existentes no processo de vacinação em geral e, especificamente, no caso da vacinação contra a *Mpox* em PVHA. A equipe de enfermagem desempenha um papel direto na organização e implementação das ações de vacinação. Além disso, possibilita perspectivas de planejamento focado para gestores e profissionais de saúde, de modo que seja possível a implementação de intervenções eficazes para atingir todo o público-alvo, garantindo o acesso universal, igualitário e equitativo à vacinação e, consequentemente, reduzindo a formação de grupos suscetíveis e a morbimortalidade associada à doença. Isso terá um impacto direto no cenário epidemiológico brasileiro.

## CONCLUSÕES

Identificamos baixas coberturas vacinais para as PVHA em todas as regiões do Brasil. Entre elas, a região sul foi a que apresentou as menores coberturas vacinais, e os estados do Rio Grande do Norte, Mato Grosso e Espírito Santo registraram uma cobertura igual a zero. Além disso, observa-se a necessidade de intensificar as atividades de vacinação, principalmente para as PVHA, e também de aumentar a adesão de ambos os sexos para completar o esquema vacinal, a fim de evitar a formação de grupos suscetíveis. É provável que a disponibilização de outros dados pelo sistema permitirá uma ampliação na avaliação dos dados, incluindo novos indicadores para analisar outras variáveis relacionadas à situação vacinal das PVHA no Brasil.

É importante destacar que este estudo possibilitou uma análise crítica e detalhada da cobertura vacinal nas diferentes regiões do país, bem como destacou a necessidade de aprimorar políticas públicas que atendam às mudanças epidemiológicas ocorridas e promovam melhores condições de vida e saúde para essa população, contribuindo assim para minimizar os danos causados por essa doença. Considerando a perspectiva da cobertura vacinal, é fundamental a implementação de ações voltadas para a ampliação da assistência às PVHA, fornecendo subsídios para uma discussão sobre a prática assistencial relacionada à vacinação e suas peculiaridades no contexto das populações vulneráveis, visando não apenas a prevenção, mas também a melhoria da qualidade da assistência.
